# RETRACTED: Aldawsari et al. Lipidic Nano-Sized Emulsomes Potentiates the Cytotoxic and Apoptotic Effects of Raloxifene Hydrochloride in MCF-7 Human Breast Cancer Cells: Factorial Analysis and In Vitro Anti-Tumor Activity Assessment. *Pharmaceutics* 2021, *13*, 783

**DOI:** 10.3390/pharmaceutics16020195

**Published:** 2024-01-30

**Authors:** Hibah M. Aldawsari, Osama A. A. Ahmed, Nabil A. Alhakamy, Thikryat Neamatallah, Usama A. Fahmy, Shaimaa M. Badr-Eldin

**Affiliations:** 1 Department of Pharmaceutics, Faculty of Pharmacy, King Abdulaziz University, Jeddah 21589, Saudi Arabia; haldosari@kau.edu.sa (H.M.A.); oaahmed@kau.edu.sa (O.A.A.A.); nalhakamy@kau.edu.sa (N.A.A.); smbali@kau.edu.sa (S.M.B.-E.); 2 Center of Excellence for Drug Research and Pharmaceutical Industries, King Abdulaziz University, Jeddah 21589, Saudi Arabia; 3 Mohamed Saeed Tamer Chair for Pharmaceutical Industries, King Abdulaziz University, Jeddah 21589, Saudi Arabia; 4 Department of Pharmacology and Toxicology, Faculty of Pharmacy, King Abdulaziz University, Jeddah 21589, Saudi Arabia; taneamatallah@kau.edu.sa; 5 Department of Pharmaceutics and Industrial Pharmacy, Faculty of Pharmacy, Cairo University, Cairo 11562, Egypt

The journal retracts the article “Lipidic Nano-Sized Emulsomes Potentiates the Cytotoxic and Apoptotic Effects of Raloxifene Hydrochloride in MCF-7 Human Breast Cancer Cells: Factorial Analysis and In Vitro Anti-Tumor Activity Assessment” [1] cited above.

Following publication, concerns were brought to the attention of the publisher regarding the overlap of images across a number of publications.

Adhering to our complaints procedure, an investigation was conducted by the Editorial Office and Editorial Board that confirmed the overlap of Figure 4 published in [1] with Figure 8 of [2], Figure 7 of [3], Figure 7 [4], Figure 7 of [5], Figure 7 of [6], and Figure S2 of [7], listing different experimental conditions. 

While the authors fully cooperated with the Editorial Office during the investigation, they were unable to satisfactorily explain the overlap of figures presenting different experimental conditions and meet the required quality standards of raw images to consider a correction as per the journal’s original image requirements policy (https://www.mdpi.com/journal/pharmaceutics/instruc-tions#oriimages). As a result, the Editorial Board and Editor-in-Chief were unable to confirm the reliability of the findings and subsequently decided to retract this paper as per MDPI’s retraction policy (https://www.mdpi.com/ethics#_bookmark30). 

This retraction was approved by the Editor-in-Chief of the journal *Pharmaceutics*. 

The authors did not agree to this retraction. 
